# Senescence Marker Protein 30 (SMP30) Expression in Eukaryotic Cells: Existence of Multiple Species and Membrane Localization

**DOI:** 10.1371/journal.pone.0016545

**Published:** 2011-02-09

**Authors:** Peethambaran Arun, Vineela Aleti, Kalpana Parikh, Veeraswamy Manne, Nageswararao Chilukuri

**Affiliations:** Department of Molecular Pharmacology, Division of Biochemistry, Walter Reed Army Institute of Research, Silver Spring, Maryland, United States of America; University of South Florida College of Medicine, United States of America

## Abstract

Senescence marker protein (SMP30), also known as regucalcin, is a 34 kDa cytosolic marker protein of aging which plays an important role in intracellular Ca^2+^ homeostasis, ascorbic acid biosynthesis, oxidative stress, and detoxification of chemical warfare nerve agents. In our goal to investigate the activity of SMP30 for the detoxification of nerve agents, we have produced a recombinant adenovirus expressing human SMP30 as a fusion protein with a hemaglutinin tag (Ad-SMP30-HA). Ad-SMP30-HA transduced the expression of SMP30-HA and two additional forms of SMP30 with molecular sizes ∼28 kDa and 24 kDa in HEK-293A and C3A liver cells in a dose and time-dependent manner. Intravenous administration of Ad-SMP30-HA in mice results in the expression of all the three forms of SMP30 in the liver and diaphragm. LC-MS/MS results confirmed that the lower molecular weight 28 kDa and 24 kDa proteins are related to the 34 kDa SMP30. The 28 kDa and 24 kDa SMP30 forms were also detected in normal rat liver and mice injected with Ad-SMP30-HA suggesting that SMP30 does exist in multiple forms under physiological conditions. Time course experiments in both cell lines suggest that the 28 kDa and 24 kDa SMP30 forms are likely generated from the 34 kDa SMP30. Interestingly, the 28 kDa and 24 kDa SMP30 forms appeared initially in the cytosol and shifted to the particulate fraction. Studies using small molecule inhibitors of proteolytic pathways revealed the potential involvement of β and γ-secretases but not calpains, lysosomal proteases, proteasome and caspases. This is the first report describing the existence of multiple forms of SMP30, their preferential distribution to membranes and their generation through proteolysis possibly mediated by secretase enzymes.

## Introduction

Senescence marker protein 30 (SMP30) was identified from rat liver in 1992 as an aging factor, the expression of which decreases with age in an androgen independent manner suggesting its possible roles in age related physiologic and pathologic conditions [1]–[3]. Regucalcin was known since 1978 as a calcium-binding protein without the typical Ca^2+^ binding EF-motif and has been extensively studied for its role in the maintenance of Ca^2+^ homeostasis and Ca^2+^ signaling in rat liver and kidney cells [4]–[7]. Following the cloning and characterization of genes encoding these proteins, it became clear that SMP30 and regucalcin are one and the same with 299 amino acids and an estimated molecular weight of 33387 Daltons [2]–[8]. Nonetheless, there appears to be no consensus on the nomenclature for this protein and we use SMP30 in our manuscript. SMP30 has a highly conserved structure across various animal species [9], [10] and is widely distributed in different tissues including liver, kidney, brain, testis, lungs, adrenal gland, stomach, ovary, uterus and epidermis [11]. Immunohistochemical and western blot analysis shows that SMP30 is localized in the cytosol and nucleus of hepatocytes [12] and in the case of kidney, the immunoreactivity was primarily in renal proximal tubular epithelia [2].

The reported functions and activities of SMP30/regucalcin are varied. One of the major roles described for SMP30 is in maintaining Ca^2+^ homeostasis by activating enzymes involved in the regulation of Ca^2+^ pump localized in the plasma membrane, microsomes and mitochondria of different cell types [5]. SMP30 can bind to Ca^2+^ even though it lacks the known Ca^2+^ binding motif such as EF-hand [13]. In the nucleus, SMP30 is believed to be involved in the regulation of protein kinases, protein phosphatases and deoxyribonucleic acid and ribonucleic acid biosynthesis [5]. Over expression of SMP30 in rats leads to osteoporosis [14] and hyperlipidemia [15] while SMP30 deficiency in mice causes accumulation of neutral lipids and phospholipids in the liver [16] showing its critical roles in bone and lipid metabolism. Studies conducted using SMP30 knock-out mice indicate that brain SMP30 has a protective role against oxidative damage without influencing the enzymes involved in antioxidant protection [17]. SMP30 also possesses gluconolactonase activity and hence play an important role in ascorbic acid biosynthesis in the liver [18].

Our interest in SMP30 grew out of three studies which reported that SMP30 and/or a structurally related protein from mouse and rat livers hydrolyzed disiopropylfluorophosphate (DFP) and chemical warfare nerve agents including soman, sarin, VX, and tabun [19]–[21]. In addition, SMP30 knock-out mice lacked DFPase activity implying that SMP30 may be the DFP hydrolyzing enzyme in the liver and hence it can be a potent catalytic bioscavenger against nerve agents [19]. Even though there are structural similarities between SMP30 and serum paraoxonase1 (PON1), another potential catalytic bioscavenger, the inability of SMP30 to hydrolyze PON1 specific substrates makes SMP30 distinct from PON family [19].

Mitigating the risk posed by the potential use of nerve agents in warfare as well as in civilian populations is strategic and high priority. In order to determine whether human SMP30 functions in the hydrolysis of nerve agents *in vivo*, we have produced a recombinant adenovirus expressing human SMP30 as a fusion protein with a hemaglutinin tag (Ad-SMP30-HA). In this manuscript, we present novel observations from Ad-SMP30-HA transduced expression of SMP30-HA in human embryonic kidney epithelial cells (HEK-293A) and hepatoma carcinoma (C3A) cells. In addition to the 34 kDa intact SMP30, two additional forms of SMP30 with molecular sizes of ∼28 kDa and ∼24 kDa were consistently and reproducibly detected in cell extracts raising the possibility that the virus-expressed SMP30 underwent intracellular processing. While a significant portion of 34 kDa SMP30 in cells was cytosolic, a substantial majority of the protein was also localized to crude mitochondrial fraction and microsomal membranes. On the other hand, the 28 kDa and 24 kDa lower molecular weight forms appeared initially in the cytosol and shifted to the particulate fraction. All the three forms of SMP30-HA were expressed in the liver and diaphragm of mice injected with Ad-SMP30-HA. The 28 kDa and 24 kDa SMP30 forms were also detected in normal rat liver suggesting that native enzyme also undergoes a similar processing. Inhibitors of calpains, lysosomal proteases, the proteasome and caspases had no effect on SMP30 processing. However, inhibitors specific to both β and γ-secretases inhibited the expression of smaller SMP30 forms suggesting their possible involvement in the processing of SMP30.

## Results

### Expression and dose response of Ad-SMP30 in HEK-293A and C3A cells

**Figure 1:**
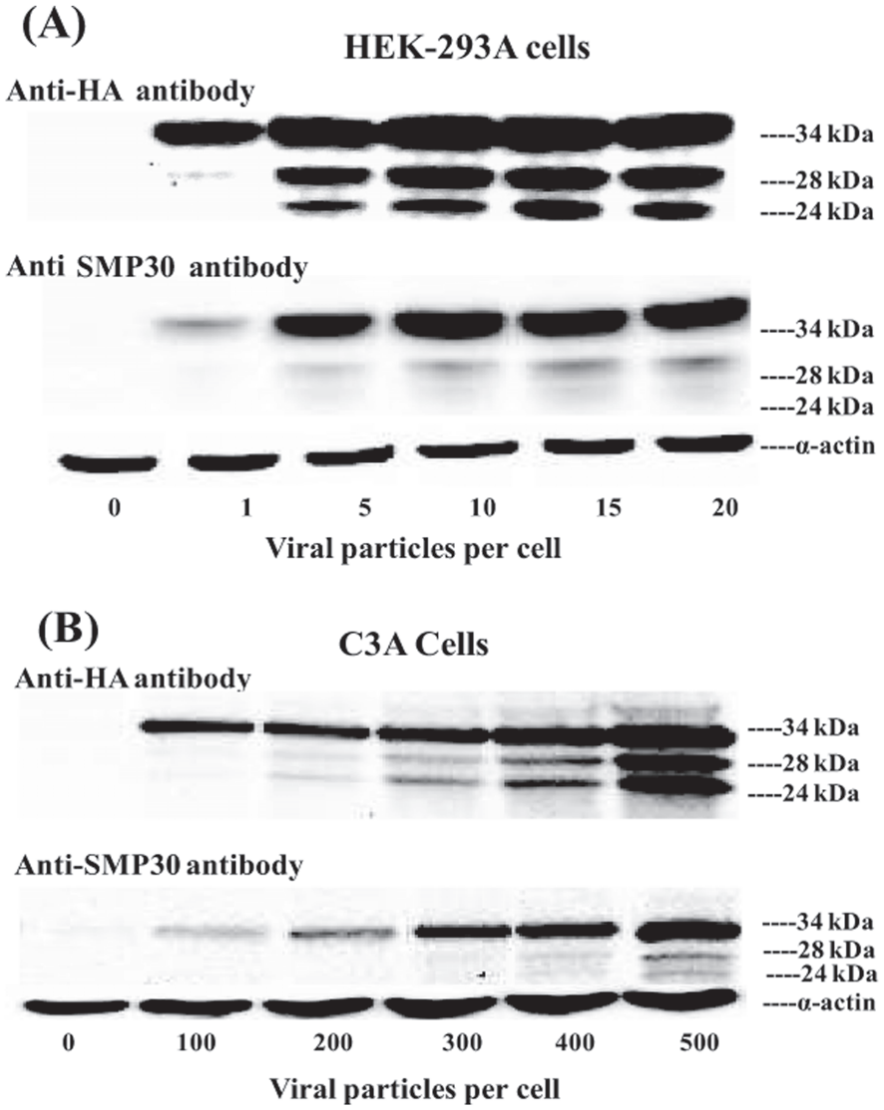
Dose response of Ad-SMP30-HA in HEK-293A cells and C3A liver cells. HEK-293A cells (A) and C3A liver cells (B) were treated with the indicated number of viral particles for 72 h and the expression of SMP30-HA was analyzed by western blotting using SMP30 or HA antibodies.

Figure 1 shows the expression of SMP30 in HEK-293A cells (A) and in C3A liver cells (B). In addition to the expected 34 kDa protein, two additional low molecular weight proteins (∼28 kDa and 24 kDa) were also detected using the antibodies against both HA-tag and native SMP30 protein. Expression of SMP30 increased as the VP/cell for infection was raised in both the cell lines. Maximal expression was achieved with 10–20 VP/cell in 293A cells and required 20 times higher number in C3A liver cells since the virus does not multiply in C3A cells because these cells lack the E1 gene products which are required for multiplication of the virus. E1 gene products are expressed in HEK-293A cells. The presence or accumulation of SMP30 related low molecular weight proteins was optimal with viral particles producing maximal SMP30 expression in both the cell lines. These results suggest that our virus was biologically active and transduced the expression of SMP30 in a dose dependant manner. Immunoreactivity of the low molecular weight proteins to both the antibodies suggest that they are likely related to the 34 kDa SMP30 protein. All the three forms of SMP30 were observed on a consistent basis and repeatedly when we took extreme precautions including quick and direct lysis into SDS-PAGE sample buffer and making extracts in the presence of a cocktail of protease inhibitors (results not shown). Actin controls were used to ascertain equal sample loading across various lanes.

### Confirmation of the identity of SMP30-HA and its processed forms using LC-MS/MS

**Figure 2:**
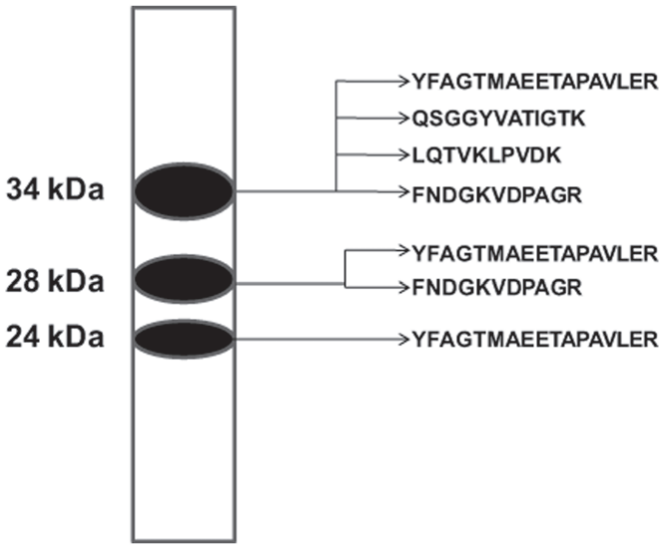
Schematic diagram showing the gel areas subjected to LC-MS/MS and peptides recovered therefrom.

We explored the three different forms of SMP30 further by nano-flow LC-MS/MS after purifying partially by HA-affinity chromatography. Using a SMP30 western blot as a guide, the three protein bands from an SDS-PAGE gel were cut out separately as indicated in Figure 2. Following the processing of the gel pieces through standard procedures for nano-flow LC-MS/MS, analysis of the data from protein bands corresponding to SMP30 and its lower molecular weight forms showed that all the three proteins carried the amino acid sequence of human SMP30. The 34 kDa protein band yielded four peptide sequences of human SMP30: YFAGTMAEETAPAVLER, FNDGKVDPAGR, LQTVKLPVDK and QSGGYVATIGTK; the 28 kDa protein band produced YFAGTMAEETAPAVLER and FNDGKVDPAGR; and the 24 kDa protein band gave YFAGTMAEETAPAVLER. These LC-MS/MS results confirm that the lower molecular weight proteins are related to 34 kDa SMP30 protein and were likely formed from it.

### Presence of low molecular weight forms of SMP30 in normal rat liver

**Figure 3:**
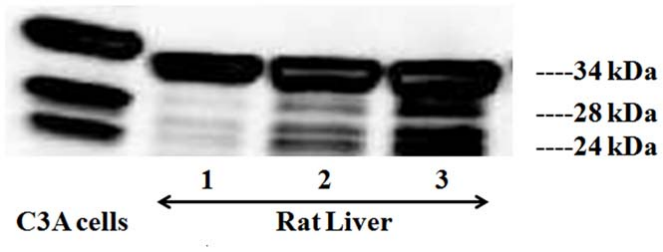
Presence of multiple forms of SMP30 in 7 day old normal rat pup. Rat liver cytosolic extract in increasing amounts (10, 20 and 30 µg total protein marked as 1, 2 and 3) were subjected to western blotting using antibody against SMP30. Extracts of C3A liver cells expressed SMP30-HA was shown for comparison.

Since SMP30 has not been reported to exist in multiple forms and to rule out the possibility that the lower molecular weight forms are an artifact of very high levels of gene expression, we explored for their existence in rat tissues. We chose young rat livers that are known to make high levels of SMP30. Western blot analysis of the liver extracts from 7 day old rat pup using the antibody against SMP30 identified the 34 kDa form as the predominant species. In addition, we also detected the three additional lower molecular weight forms of SMP30, particularly visible in lanes 2 and 3 loaded with large amounts of proteins (Figure 3). Two lower size forms are comparable to 24 kDa and 28 kDa forms found in C3A liver cells infected with Ad-SMP30-HA (Figure 3).

### Presence of low molecular weight SMP30 forms in the liver and diaphragm of mice injected with Ad-SMP30-HA

**Figure 4:**
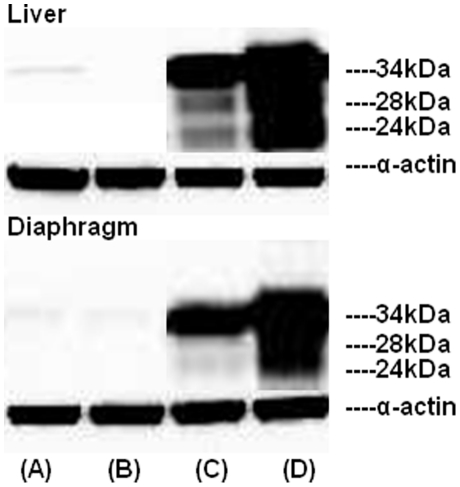
Presence of multiple forms of SMP30 in the liver and diaphragm of mice injected with 2×10^11^ Ad-SMP30-HA per animal. The animals were injected with the control virus (Ad-null, A & B) or Ad-SMP30-HA (C & D) via the tail vein at a dose of 2×10^11^ VP/animal and sacrificed on day 4 post-virus injection. Tissue homogenates were processed by western blotting using anti-HA antibody.

We investigated whether the Ad-SMP-30 is biologically active *in vivo* in mice and whether the 28 kDa and 24 kDa forms of SMP30 are also formed *in vivo* following virus injection in mice. Mice were injected with Ad-SMP30-HA or a control virus and 4 days later, liver and diaphragm were processed for the presence of various forms of SMP30 by western blotting using anti-HA antibody. The 28 kDa and 24 kDa SMP30 forms were detected in both tissues suggesting that human SMP30 does undergo processing and exists in multiple forms *in vivo* (Figure 4). No detectable amount of the full length SMP30-HA or the processed forms were detected in the liver and diaphragm of mice injected with a control virus.

### Localization of SMP30 and its lower molecular weight proteins

**Figure 5:**
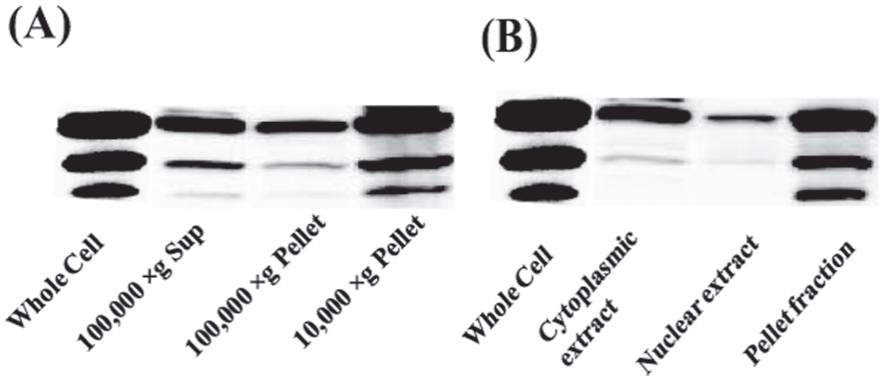
Subcellular localization of SMP30 forms in HEK-293A cells 72 h following the infection with Ad-SMP30-HA (20 VP/cell). A, Fractions obtained by centrifugation; B, Fractions derived using NE-PER nuclear and cytoplasmic extraction kit. The amounts of various forms from different fractions shown in A & B represent the actual amounts shown to be present in the total whole cell lysate. Anti-HA antibody was used to probe the western blots.

Mouse SMP30 has been detected previously in the cytoplasm, nucleus and mitochondria, but there were no reports of the existence of multiple forms of SMP30 and their distribution. To gain a better understanding of the various forms of human SMP30 detected in our system and their sub-cellular distribution, we fractionated Ad-SMP30-HA infected HEK-293A cell extracts into cytosolic, microsomal and particulate fractions. The results in Figure 5A show that while a significant portion of 34 kDa SMP30 protein in HEK-293A cells was cytosolic, a substantial majority of the protein was also localized to crude mitochondrial (10,000×g pellet) and microsomal membrane (100,000×g pellet) fractions. Both of the lower molecular weight forms of SMP30 were also detectable in all fractions, albeit to varying degrees. Nonetheless, the results suggest that the lower molecular weight forms of SMP30 are localized predominantly in the particulate fraction, particularly mitochondria. The particulate association of these multiple SMP30 forms is quite intriguing and merit further study. We also determined the nuclear distribution of various SMP30 forms using NE-Per nuclear and cytoplasmic extraction kit from Pierce Biotechnology according to manufacturer's instructions. The results shown in Figure 5B suggest that ∼10% of the total 34 kDa SMP30 was nuclear in localization and the lower molecular weight forms were also present, but barely detectable. The amounts and distribution of different SMP30 forms in the cytosolic or particulate fractions using the NE-Per extraction kit are in excellent agreement with the results obtained above from classical fractionation by centrifugation.

### Time course expression of SMP30-HA and its small molecular weight forms

**Figure 6:**
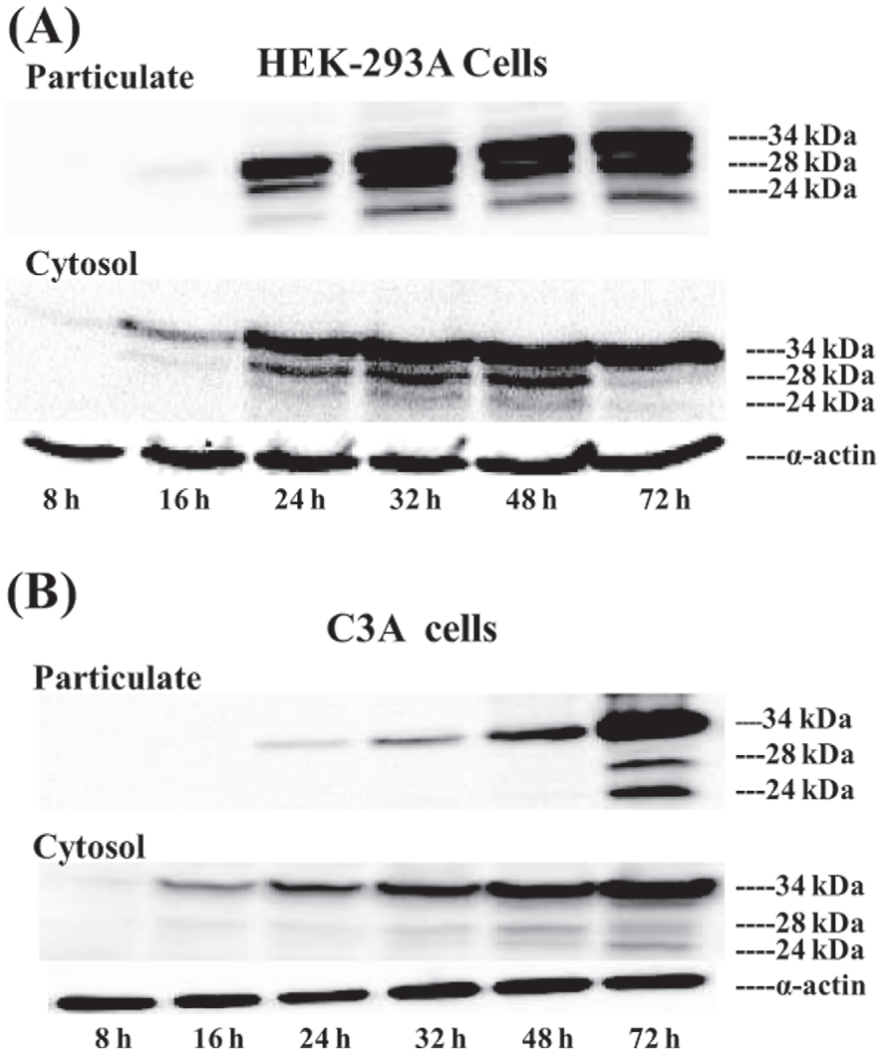
Time course expression of SMP30 and its processed forms in HEK-293A cells and C3A cells. HEK-293A cell (A) and C3A cells were treated with Ad-SMP30-HA (20 VP/cell for HEK-293A cells and 500 VP/cell for C3A liver cells) for varying periods of time, cell extracts were prepared in M-PER buffer and separated into the particulate and cytosolic fractions. Western blotting was carried out using antibody against HA tag.

It is not clear whether the low molecular weight SMP30 proteins are precursors or products of the larger 34 kDa SMP30 protein. However, the former possibility is unlikely since the presence of optimal levels of the low molecular weight proteins correlated with maximal expression of 34 kDa SMP30 species (Figure 1). To support these observations, the biosynthesis and distribution of SMP30 and its various forms was followed over a period of 72 h in HEK-293A and C3A liver cells after infection with Ad-SMP30-HA (Figure 6). Both the cell lines gave similar results. The 34 kDa SMP30 initially appeared as early as 8 h in the cytosolic fraction and gradually increased over the examined time period of 72 h. Interestingly, near equal amounts of the 34 kDa form accumulate in the cytosol and particulate fraction as peak levels of SMP30 are made after 24–48 h. The results also indicate that the lower molecular weight forms begin to appear initially in the cytosol over 16–24 h time period and gradually shift to the particulate fraction, with the shift near complete by 72 h. The kinetics and distribution observed in both the cell lines suggest that the smaller molecular weight proteins are likely derived from the 34 kDa protein and preferentially associate with the particulate (mitochondria) fraction.

### Effect of small molecule inhibitors on the processing of SMP30

**Figure 7:**
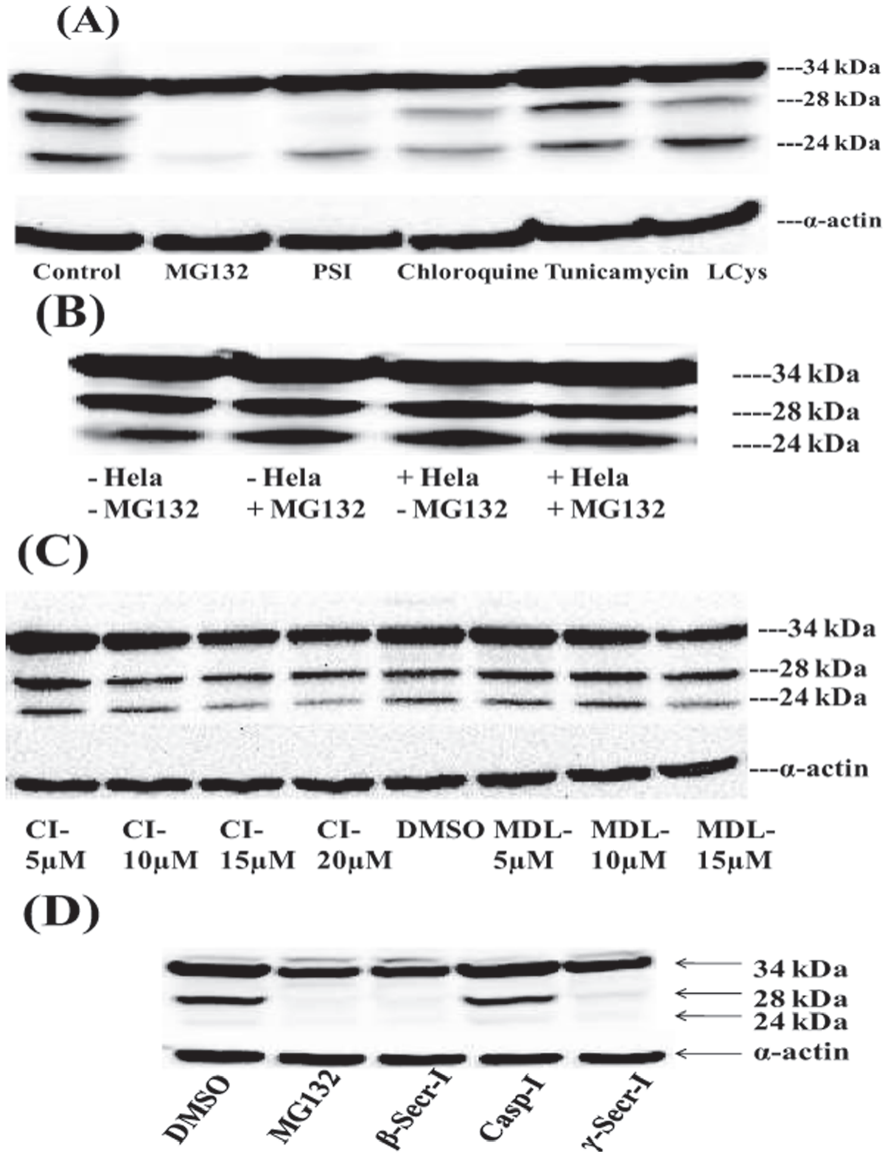
Effect of protease inhibitors on the expression and processing of SMP30 in HEK-293A cells. A, The inhibitors, MGI32 (10 µM), PSI (50 µM), LCys (10 µM), chloroquine (50 µM) and tunicamycin (3 µg/ml) were added along with Ad-SMP30-HA (20 VP/cell) to the cells and incubated for 24 h; B, Effect of proteasomal degradation system on the processing of SMP30 protein expressed in HEK-293A cells in the presence or absence of 10 µM MG132; C, Varying concentrations of calpain inhibitor I (CI, 5, 10, 15 and 20 µM) or MDL28170 (MDL, 5, 10 and 15 µM) were added to the cells along with Ad-SMP30-HA (20 VP/cell) and incubated for 24 h; D, HEK-293A cells were treated with 20 µM caspase inhibitor I [Z-VAD(OMe)-FMK] or 5 µM β-secretase inhibitor (Z-VLL-CHO) or 5 µM γ-secretase inhibitor (Z-LLNle-CHO) or 10 µM MG132 along with Ad-SMP30-HA (20 VP/cell) for 24 h; Equal volume of DMSO served as control. The cells were harvested, lysed with SDS-PAGE buffer containing 5% β-mercaptoethanol and SDS-PAGE followed by western blotting was carried out using anti-HA antibody.

Proteasomal and lysosomal mechanisms are two classical protein degradation systems and are widely used in cells. We investigated the potential involvement of these and other proteolytic pathways in SMP30 processing by making use of small molecule inhibitors of proteolysis. Figure 7A–D shows the results obtained when SMP30-HA expression was carried out in the presence of various inhibitors. The processing of SMP30-HA was unaffected by chloroquine, a lysosomal inhibitor or tunicamycin, a glycosylation inhibitor (Figure 7A). Lactacystin, an irreversible specific inhibitor of the proteasome, also did not affect the processing of SMP30-HA (Figure 7A). However, MG132 and PSI, which are potent reversible non-specific inhibitors of proteasome, significantly inhibited the processing of SMP30-HA (Figure 7A). Since the results obtained with various inhibitors of proteasome are conflicting, we conducted experiments to demonstrate the involvement of proteasome in SMP30 processing directly using Hela cell S-100 degradation mixture. The S-100 Hela proteasomal degradation mixture contains the full complement of ubiquitin proteasome pathway enzymes necessary for the controlled degradation of proteins. When Hela cell S-100 mixture was used to treat 293A cell made SMP30 proteins, the processing of 34 kDa SMP30 protein was not affected either in the presence or absence of MG132 (Figure 7B). These results suggest that the processing of SMP30 protein is unlikely to be a result of the ubiquitin-proteasome degradation pathway. We also tested the possible involvement of calpain family of calcium-dependent, non-lysosomal cysteine proteases in SMP30 processing by using calpain inhibitor I and MDL28170. Both, known potent inhibitors of calpains, did not affect the processing of SMP30-HA (Figure 7C) which suggests that the processing of SMP30 is mediated through proteolytic enzymes outside of the calpains. We also found that the processing of SMP30 protein was unaffected by inhibitor of caspases (Figure 7D), thus ruling out the possible involvement of caspases in the proteolytic processing of 34 kDa SMP30.

The lack of effect of inhibitors of a broad range of proteases including calpains, lysosomal proteases, the proteasome and caspases is surprising in view of the reported existence of PEST motifs in the amino acid sequence of SMP30 [2]. However, we did observe that Inhibitors of both β and γ-secretases blocked the processing of SMP30, with the former showing maximum effect (Figure 7D). While these results suggest that secretases may be somehow involved in the processing of SMP30, extensive studies are required to address how the apparently cytosolic SMP30 could be processed by membrane buried secretases.

## Discussion

In this study, for the first time, we present evidence that the full-length 34 kDa SMP30 protein undergoes intracellular processing to produce two additional forms of SMP30 with molecular sizes of ∼28 kDa and 24 kDa. Two lines of evidence support that the 28 kDa and 24 kDa proteins are related to 34 kDa SMP30: 1) polyclonal antibodies to native 34 kDa SMP30 protein reacts with 28 kDa and 24 kDa proteins suggesting the three proteins share common antigenic epitopes, and 2) all three proteins contain one or more peptides with identical amino acid sequences. Our results also suggest that the processing of SMP30 happens close to the N-terminus while the C-terminus of the protein stays intact. This is based on the observation that the C-terminal HA-tag is recognized by HA antibodies in both the 28 kDa and 24 kDa forms of SMP30. Also, a peptide with amino acid sequence “YFAGTMAEETAPAVLER” that is 113 amino acids away from the N-terminus is present in all three forms of SMP30. Our data also suggest that the 28 kDa and 24 kDa forms tend to associate with particulate (mitochondria) fraction in cells whereas the full-length 34 kDa SMP30 is equally distributed between the cytosolic and particulate fractions. The 28 kDa and 24 kDa SMP30 forms were also detected in normal rat liver suggesting that SMP30 does exist in multiple forms under physiological conditions. Moreover, injection of Ad-SMP30-HA into mice resulted in the production of 28 kDa and 24 kDa lower molecular weight SMP30 forms in addition to the 34 kDa protein in the liver and diaphragm suggesting that the existence of a processing mechanism for 34 kDa SMP30 in mice. The identity of an additional band observed (using western blotting with anti-SMP30 antibody) in liver extracts of normal 7 day old rat is unclear at present, but it was absent (using western blotting with anti-HA antibody) in liver extracts of Ad-SMP30-HA injected mice suggesting the possibility that the anti-SMP30 antibody may have non-specific cross-reactivity.

Stable and high level expression of SMP30/regucalcin has been demonstrated in several eukaryotic cell lines that include human hepatocellular carcinoma Hep G2, Rat hepatoma H4-II E, mouse embryonic carcinoma PC19 and normal rat kidney proximal tubular epithelial NRK 52E [10], [22]–[26]. The levels of expression obtained in these studies ranged from 5 to 20-fold over the vector controls and our results with adenovirus vector achieved much higher expression of >100-fold over the vector controls in HEK-293A cells (Figure 1 & 6). None of the above studies reported the formation or existence of multiple forms of SMP30. While the reasons for the discrepancy are not completely clear at present, all the three forms were observed in our studies on a consistent basis and repeatedly in both the cell lines used and *in vivo* in mice following Ad-SMP30-HA injection. Some of these studies may have used the high speed centrifuged cytosolic fractions for western blot analysis and thus missed the other species which tend to associate preferentially with particulate fractions (see later in discussion). Also, one of these studies expressed SMP30 with HA tag at the N-terminus [26] and thus could have missed the processed forms. In any case, we used LC-MS/MS and polyclonal antibodies to SMP30 to confirm that the lower molecular weight proteins are related to the 34 kDa SMP30 protein.

Our sub-cellular fractionation studies show that while a significant portion of 34 kDa SMP30 in 293A cells was cytosolic, a substantial part of the protein was also localized to the crude mitochondrial fraction and microsomal membranes. On the other hand, both the lower molecular weight forms of SMP30 proteins were found predominantly in the particulate fraction, particularly mitochondria. The kinetics and distribution observed in both the cell lines used suggest that the smaller molecular weight proteins are likely formed from the 34 kDa protein in the cytosol and preferentially migrate to mitochondria and microsomal membrane. This is consistent with a report showing regucalcin (SMP30) was localized in the mitochondria of normal rat heart and functions to regulate Ca^2+^-ATPase activity [27]. These results raise several intriguing questions for further study. Since SMP30 is known to be involved in the regulation of Ca^2+^-homeostasis by influencing the enzymes involved in Ca^2+^ pump in the plasma membrane, microsomal and mitochondrial membranes [5], it is tempting to speculate for distinct yet unknown functions for the processed forms of SMP30 in the membrane.

Several studies have reported the presence of SMP30 in the nucleus [11], [12], [28] where it was demonstrated to have a role in the regulation of nuclear function through Ca^2+^ -dependent kinases, GTPases and protein phosphatases [29]–[32]. Recombinant SMP30 produced in HEK-293A cells in our studies was also present in the nucleus. Extraction of nuclear proteins using NE-Per nuclear and cytoplasmic extraction kit from Pierce Biotechnology suggested that ∼10% of the total SMP30 is nuclear in HEK-293A cells (Figure 5B).

Proteolysis is a cellular mechanism not only for maintaining the concentration of proteins but also for regulating or modifying the functions of proteins [33], [34]. The major proteolytic processing pathways involve ubiquitin-proteasome, calpains, caspases and lysosomal proteases. Limited or controlled proteolysis through cleavage of the protein at unique amino acid sequences is a mechanism to modify proteins for different functions. Several sequence motifs which signal either sensitivity to proteolysis or entry into degradation pathways have been determined [35]. One of those sequences called PEST sequence (sequence rich in proline, glutamic acid or aspartic acid, serine and threonine) is involved in targeting proteins for rapid as well as highly conditional site specific cleavage [35], [36]. One moderately strong (amino acids 15–27) and at least four weak PEST regions (amino acids 51–64, 112–129, 130–141 and 163–190) have been identified in the amino acid sequence of SMP30 [2] which suggest that SMP30 is vulnerable to proteolytic degradation. Based on ∼24 kDa and ∼28 kDa molecular sizes of the processed SMP30 forms, we project that the sites of proteolytic cleavage are upstream of amino acids 80, an area in close proximity to the PEST regions. MG132 and PSI, which are known reversible non-specific inhibitors of proteasome, inhibited the processing of SMP30, while the proteasome specific irreversible inhibitor, LCys, had no effect on SMP30 processing in HEK-293A cells. These conflicting results were reconciled by the demonstration that SMP30 processing was unaffected by S-100 Hela degradation mixture, which contains all reagents necessary for degradation of proteins through the ubiquitin-proteasome pathway. As for the inhibition observed with MG132 and PSI, it is notable that MG132 effects in cells are not specific to the proteasome and include several other targets including the endoplasmic reticulum [37]. Also, MG132 can nonspecifically inhibit secretases which are known to be involved in the proteolytic processing of various proteins [38]. Based on these results we conclude that the processing of SMP30 is not mediated through the ubiquitin-proteasomal pathway.

Lysosomal proteases are also involved in the degradation of proteins not only to inactivate proteins but also for modifying the functions of some proteins [39]. Chloroquine, an inhibitor of lysosomal proteases, did not affect the processing of SMP30, which excludes the involvement of lysosomes on the processing of SMP30. Glycosylation processes also do not appear to be involved during the processing of SMP30 since tunicamycin did not show any inhibition. Calpains are another family of proteolytic enzymes containing ubiquitous and tissue-specific isoforms of Ca^2+^-activated cysteine proteases which can modify the functions of proteins by cleaving at a limited number of specific sites [40]. Processing of SMP30 was unaffected by calpain inhibitor I and MDL28170, which are potent inhibitors of calpains suggesting the possibility of a calpain independent proteolytic processing of SMP30.

Even though inhibitors to both β and γ-secretases blocked the processing of SMP30, disparate compartmentalization of SMP30 and secretases preclude us to suggest that secretases are directly responsible for the processing of SMP30. Detailed further studies are warranted not only for demonstrating the specific proteases and cleavage sites involved in the processing of SMP30 but also for understanding the physiological function(s) of the smaller forms of SMP30. In addition, the potential involvement of alternative splicing in generating the 28 kDa and 24 kDa SMP30 forms must be thoroughly investigated using more sensitive methods such as random hexamer reverse transcription semi-nested PCR or exon specific reverse transcription semi-nested PCR [41] even though an earlier study found no evidence of alternative splicing for SMP30 in the coding region [42] by rapid amplification of cDNA ends (RACE).

## Materials and Methods

### Chemicals and reagents

Lactacystin (LCys), MG132, tunicamycin, chloroquine, calpain inhibitor I, MDL28170, α-actin antibody, protease inhibitor cocktail, EZview red HA-affinity gel and HA-peptide were obtained from Sigma-Aldrich (St. Louis, MO). EMD Biosciences (Gibbstown, NJ) served as the source for proteasomal Inhibitor I (PSI) caspase inhibitor I [Z-VAD (OMe)-FMK], β-secretase inhibitor (Z-VLL-CHO) and γ-secretase inhibitor (Z-LLNle-CHO). Boston Biochem (Cambridge, MA) supplied the S-100 Hela degradation mixture. SMP30 antibody was supplied by Cosmo Bio Co. (Denver, CO) and HA-probe antibody was from Santa Cruz Biotechnology (Santa Cruz, CA). HEK-293A cells were from MP Biomedicals/QBiogene Inc (Irvine, CA) and C3A liver cells from ATCC (Manassas, VA).

### Production of recombinant adenovirus expressing human SMP30

Full-length human SMP30 cDNA was obtained from human liver first-strand cDNA (Origene Inc., Rockville, MD) by PCR using pfu polymerase (Stratagene, La Jolla, CA) and primers that can amplify SMP30 coding sequences from start to stop codon. The primer sequences were 5′-ATG TCT TCC ATT AAG ATT GAG TG-′3 (forward) and 5′-TCA CCC GCA TAG GAG TAG GGA-3′ (reverse). The amplified cDNA products (900 bases long) were purified and subcloned into pCR4 Blunt-TOPO vector (Invitrogen, Carlsbad, CA). Recombinant plasmid DNA was isolated and the SMP30 cDNA insert was fully sequenced to confirm its authenticity. This plasmid DNA fused with the nucleotide sequence to code for HA-tag at its carboxyl terminus was used for making recombinant adenovirus expressing human SMP30 (Ad-SMP30-HA) as a fusion protein with HA-tag (Welgen Inc., Worcester, MA). The titer of the purified virus was 1×10^12^ virus particles (VP)/ml of which 20 to 30% were infectious particles among three different batches of the virus used in our studies.

### Cells and expression of recombinant SMP30-HA

HEK-293A cells and C3A liver cells were grown in 6-well cell culture treated plates in Dulbecco's Modified Eagle Medium supplemented with 10% fetal bovine serum and antibiotics (penicillin and streptomycin). One million cells per well were seeded on the 6-well plates one day before viral infection. For dose response study in cells, increasing number of VP/cell were added into the medium and kept for 72 h. After 72 h, the cells were lysed with 300 µl of 1×Sodium Dodecyl Sulfate (SDS) gel loading buffer containing 5% β-mercaptoethanol followed by incubating at 95°C for 10 min.

### SDS-Polyacrylamide Gel Electrophoresis (SDS-PAGE) and western blotting

SDS-PAGE was carried out with precast 10% Tris-HCl gels. After electrophoresis, the proteins were transferred to PVDF membrane (GE Healthcare, Piscataway, NJ) using a Bio-Rad transfer apparatus. The membrane was blocked in 4% powdered milk for 1 h, washed once with TTBS buffer and kept overnight in primary antibody made in 0.5% milk powder containing 0.01% sodium azide. The primary antibodies used were either rabbit-anti-HA polyclonal antibody (1∶1000 dilution) or rabbit-anti-SMP30 polyclonal antibody (1∶1000 dilution) or mouse-α-actin monoclonal antibody conjugated with Horse Radish Peroxidase (1∶50,000 dilution). The membrane was then washed with TTBS five times with intermittent shaking for 8 min and incubated with secondary antibody made in 0.5% milk powder for 1 h. The secondary antibody used was Goat-anti-rabbit conjugated with Horse Radish Peroxidase (KPL, Inc, Rockville, MD; 1∶2500 dilution). No secondary antibody was used in the case of α-actin. The membrane was washed again as above and the protein bands were detected using ECL-Plus western blot detecting reagent (GE Healthcare, Piscataway, NJ) and the chemiluminescence was measured in a Bio-Rad image reader.

### Sub-cellular localization of SMP30-HA and its processed forms

HEK-293A cells (∼50×10^6^) at peak SMP30 expression (72 h post-virus infection) were washed with ice-cold phosphate buffered saline and incubated at 4°C for 30 min in 10 mM HEPES pH 7.4 buffer containing 1 mM MgCl_2_, 1 mM EGTA, 0.1 mM EDTA and protease inhibitor cocktail. Cells were then sonicated gently and the lysate was centrifuged at 10,000×g for 10 min to obtain the mitochondrial fraction in the pellet. The supernatant was centrifuged further at 100,000×g for 60 min at 4°C. The sedimented pellet (microsomes), the supernatant (cytosol) and the mitochondrial particulate fraction were used for western blotting using anti-HA antibody. Occasionally, particulate and crude cytosolic fractions were prepared by lysis of cells into 200 µl M-Per protein extraction buffer (Pierce Chemicals, Rockford, IL) followed by centrifugation at 5000 rpm for 10 min in a microcentrifuge. Cytosol (20 µl) was used for SDS-PAGE followed by western blotting using anti-HA antibody. To the pellet fraction 200 µl of 1 x SDS-PAGE sample buffer containing 5% β-mercaptoethanol was added, incubated at 95°C for 10 min and 20 µl was used for SDS-PAGE followed by western blotting using anti-HA antibody.

### Time course expression of SMP30-HA and its processed forms in HEK-293A and C3A liver cells

Time course studies were conducted using 20 VP/cell for HEK-293A cells and 500 VP/cell for C3A liver cells. Viral particles were added to the wells of the 6-well plates containing one million cells per well and incubated at 37°C for different time periods (8, 16, 24, 32, 48 and 72 h). Following incubation, cells were harvested and particulate and cytosolic fractions were obtained using the M-Per protein extraction buffer, and proteins were separated by SDS-PAGE followed by western blotting as described above using anti-HA antibody.

### Detection of multiple forms of SMP30 in livers of normal rat pups

The experimental protocol was approved by the Animal Care and Use Committee of the Uniformed Services University of the Health Sciences (USUHS), 4301 Jones Bridge Road, Bethesda, Maryland 20814. The title of the protocol, protocol number and the PI: Studies on Canavan disease using rat and mouse models; USUHS protocol number: APG-05-025; Dr. Aryan Namboodiri. Livers from 7 day old male rat pups were homogenized using T-Per protein isolation buffer (Pierce Chemical Co, Rockford, IL). The 10% homogenate was centrifuged at 12,000×g for 10 min to collect the crude cytosolic fraction. Cytosolic proteins were passed through an Amicon micro concentrator (50 kDa molecular weight cut off, Millipore, Billerica, MA) by centrifugation at 12,000×g for 10 min to collect the flow through fraction. The resultant solution was concentrated using Amicon micro concentrator (10 kDa molecular weight cut off) by centrifugation at 12,000×g for 10 min and used for SDS-PAGE followed by western blotting using anti-SMP30 polyclonal antibody.

### Detection of multiple forms of SMP30 in the tissues of mouse injected with Ad-SMP30-HA

These studies were carried out in accordance with the guide for the Care and Use of Laboratory Animals as adopted by the U. S. National Institutes of Health. The experimental protocol was approved by the Institutional Animal Care and Use Committee of Washington Biotechnology Inc., Simpsonville, MD. The title of the protocol, protocol number and the PI: Pharmacokinetic study of SMP30 and KIAA1363 in Swiss-Webster strain mice; WBI-PK-NC-3; Dr: Nageswararao Chilukuri. All efforts were made to minimize animal suffering and to reduce the number of animals used for these studies. Adult female Swiss-Webster mice (20 to 25 g body weight, 6 to 7 weeks old, n = 10) were used for the study. Animals were given either Ad-null (control virus) or Ad-SMP30-HA (2.0×10^11^ viral particles) through the tail vein. Four days post-virus injection, the animals were sacrificed and collected tissues including liver and diaphragm for determining SMP30 expression. Ten % tissue homogenates were made in T-Per tissue protein isolation buffer (Pierce Chemical Co, Rockford, IL) containing protease inhibitor cocktail (Sigma-Aldrich, St. Louis, MO). Total protein was measured using Bio-Rad DC protein assay kit and an equal amount of protein sample was used for western blotting using anti-HA antibody as described above.

### In-gel digestion and LC-MS/MS to confirm the identity of different SMP30 forms

HEK-293A cells were infected with Ad-SMP30-HA (20 VP/cell), cultured for 48 h at 37°C, and lysed with M-Per protein isolation buffer. The lysate was centrifuged at 10,000×g for 10 min and the supernatant was collected and subjected to HA-affinity chromatography. Briefly, the cytosolic fraction was diluted 10-fold with equilibration buffer (20 mM Tris-HCl containing 0.1 M NaCl and 0.1 mM EDTA, pH 7.5) and passed through a HA-affinity column. The column was washed with wash buffer (20 mM Tris-HCl containing 1 M NaCl, pH 7.5) until the absorbance at 280 nm of wash buffer was <0.05. SMP30 and its processed forms were eluted from the column using 1 mg/mL HA-peptide in equilibration buffer. The sample was concentrated using an Amicon Centricon (10 kDa molecular weight cut-off) microconcentrator and subjected to SDS-PAGE. Area of the polyacrylamide gel containing different forms of SMP30 (identified by western blotting of a duplicate lane on the same gel) was sliced into 2 mm pieces, and digested overnight at room temperature using the *Ettan* Spot Digester (GE Healthcare, Piscataway, NJ; ITSI BioSciences, Johnstown, PA) as previously reported [43]. The plate containing the digests were evaporated to dryness, reconstituted with ultra pure water and sequenced by tandem mass spectrometry as described earlier [44]. Specifically, nano-flow LC-MS/MS was carried out with nanobore electrospray columns constructed from 360 mm o.d., 75 mm i.d. fused silica capillary with the column tip tapered to a 15 mm opening. The columns were packed with 200 A 5 µm C18 beads (Michrom BioResources, Auburn, CA) to a length of 10 cm. The mobile phase for gradient elution consisted of: a) 0.3% acetic acid, 99.7% water, and b) 0.3% acetic acid, 99.7% acetonitrile. The flow through the column was split pre-column to achieve a flow rate of 350 nl/min. All tandem mass spectra were acquired on a Thermo DECA XP Plus or LTQ ion trap mass spectrometer (Thermo Corporation, Waltham, MA) with the needle voltage set at 3 kV. Ion signals above a predetermined threshold automatically triggered the instrument to switch from MS to MS/MS mode for generating fragmentation spectra. The obtained MS/MS spectra were searched against the NCBI non-redundant protein sequence database using the SEQUEST computer algorithm [45].

### Treatment with inhibitors of proteolysis

HEK-293A cells were treated with various tool compounds at concentrations as indicated along with infection by Ad-SMP30-HA (20 VP/cell) and cultured for 24 h. Equal volume of DMSO served as control. The cells were then harvested, lysed with SDS-PAGE sample buffer containing 5% β-mercaptoethanol, analyzed by SDS-PAGE and western blotted using anti-HA antibody.

### Treatment with ubiquitin proteasome pathway enzymes using S-100 Hela degradation system

HEK-293A cells at maximal expression of SMP30 were lysed with 50 mM HEPES buffer (pH 7.6) and the supernatant was treated with S-100 Hela degradation mixture according to the manufacturer's instructions (Boston Biomedicals, Cambridge, MA). The S-100 Heal degradation mixture contains reagents to allow controlled degradation of proteins through the ubiquitin proteasome pathway. The reaction mixture was then subjected to SDS-PAGE followed by western blotting using the anti-HA antibody.
